# Identification of a novel missense mutation in the *TPM1* gene via exome sequencing in a Chinese family with dilated cardiomyopathy: A case report and literature review

**DOI:** 10.1097/MD.0000000000028551

**Published:** 2022-01-14

**Authors:** Yilong Man, Changying Yi, Meili Fan, Tianyu Yang, Peng Liu, Shiguang Liu, Guangxin Wang

**Affiliations:** a Department of Cardiology, Central Hospital Affiliated to Shandong First Medical University, Jinan, Shandong, China; b Shandong Innoviation Center of Intelligent Diagnosis, Central Hospital Affiliated to Shandong First Medical University, Jinan, Shandong, China; c Department of Clinical Laboratory, Qilu Children's Hospital of Shandong University, Jinan, Shandong, China; d Department of Acupuncture and Massage, Qilu Children's Hospital of Shandong University, Jinan, Shandong, China; e Department of Ultrasound, Central Hospital Affiliated to Shandong First Medical University, Jinan, Shandong, China; f Department of Cardiology, Jinan Central Hospital, Cheeloo College of Medicine, Shandong University, Jinan, Shandong, China.

**Keywords:** dilated cardiomyopathy, echocardiography, missense mutation, next-generation sequencing, tropomyosin 1 gene

## Abstract

**Rationale:**

Dilated cardiomyopathy (DCM) is a cardiovascular disorder characterized by consecutive ventricular dilation and contractile dysfunction, often leading to congestive heart failure. DCM type 1Y (DCM1Y) is caused by a mutation in the *TPM1* (tropomyosin 1) gene. To date, about thirty *TPM1* gene mutations have been reported to be related to DCM1Y. However, mutational screening of the *TPM1* gene is still far from being complete. Identification of *TPM1* mutation is particularly important in the diagnosis of DCM1Y and will give more insights into the molecular pathogenesis of DCM1Y.

**Patient concerns:**

A Chinese Han family with DCM phenotypes was examined.

**Diagnosis:**

A novel missense mutation, c.340G > C in exon 3 of the *TPM1* gene, was identified.

**Interventions:**

Next-generation sequencing (NGS) of DNA samples was performed to detect the gene mutation in the proband, which was confirmed by Sanger sequencing.

**Outcomes:**

This novel heterozygous mutation results in the substitution of glutamic acid with glutamine (p.E114Q). Based on this finding and clinical manifestations, a final diagnosis of DCM1Y was made.

**Lessons:**

We present evidence that p.E114Q mutation represents a novel *TPM1* mutation in a Chinese Han family with DCM. Our data expand the mutation spectrum of the *TPM1* gene and may facilitate the clinical diagnosis of DCM1Y.

## 1 Introduction

Dilated cardiomyopathy (DCM) is a cardiovascular disorder characterized by consecutive ventricular dilatation and contractile dysfunction, with an incidence of about 1 in 2500 individuals.^[1–3]^ Due to its significant prevalence, high morbidity and mortality, and the frequent hospitalization it causes, DCM is a major health issue for adults. The causes of DCM are heterogeneous, such as myocarditis, exposure to drugs, alcohol, or other toxins, and metabolic or endocrine disturbances. Genetic mutations that usually involve genes responsible for cytoskeletal, sarcomere, and nuclear envelope proteins can be identified in 30% to 40% of DCM cases.^[4,5]^ Due to the heterogeneity of DCM causes, a detailed diagnostic work-up is necessary to identify the specific underlying cause and exclude other conditions with phenotype overlap.^[6]^

Genetic studies have so far identified 42 different forms of DCM caused by mutations in more than 40 genes, such as the lamin A (*LMNA*), desmoglein 2 (*DSG2*), and tropomyosin 1 (*TPM1*) genes. Additionally, some of these genes that harbor DCM mutations are very large. Hence, examining all coding exons and intron/exon junctions for variations in multiple genes is expensive and labor-intensive. Next-generation sequencing (NGS) has been used as an alternative approach to more traditional methods for detecting gene mutations. NGS has many advantages; it not only produces massive amounts of data in parallel but also measures each base pair to an unprecedented depth, greatly reducing the time and cost of sequencing each sample at each locus.^[7]^

In this study, we described the clinical, echocardiographic, and electrocardiogram (ECG) characteristics of a Chinese Han family with DCM. We used a NGS-based method to identify a novel missense mutation, c.340G > C in exon 3 of the *TPM1* gene, in this family. Patients in this family were diagnosed as DCM type 1Y (DCM1Y).

## 2 Patients and methods

### 2.1 Proband and family investigation

**Figure 1:**
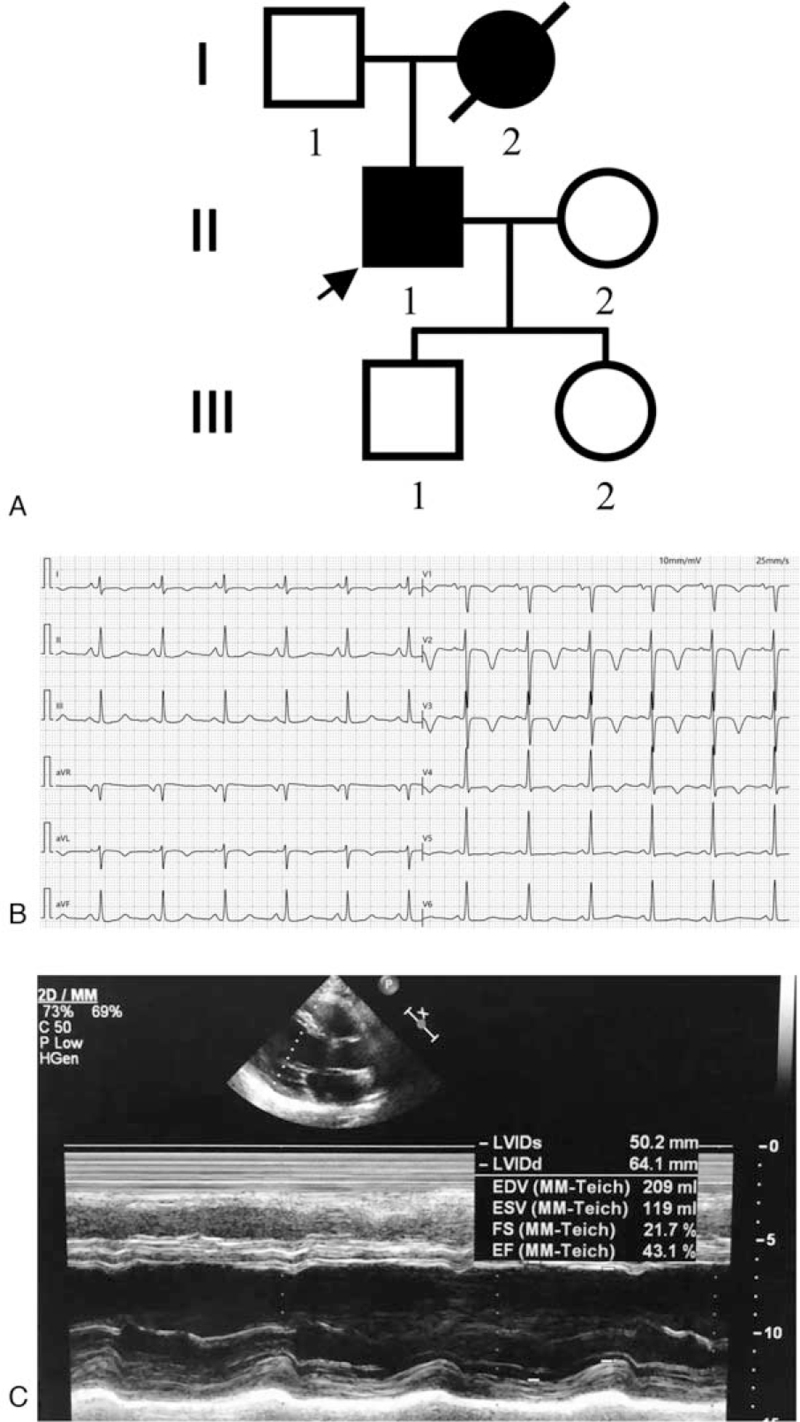
The family pedigree and examinations of the proband. (A) Pedigree of a Chinese Han family with DCM (The arrow indicates the proband). (B) The ECG shows ST-T wave changes and nodal tachycardia. (C) Echocardiography shows chamber enlargement with a left ventricular end-diastolic diameter of 33.6 mm/m^2^ and a fractional shortening of 21.7%.

Figure 1A shows the pedigree of the DCM family. The proband (II-1) was a 40-year-old Chinese Han male born at full term after an uncomplicated pregnancy and delivery. He was admitted for chest tightness and abdominal distension for 1 month. His symptoms worsened for 4 days and were accompanied by dyspnoea. His past medical history was unremarkable; he was without metabolic disorders, premature aging, or skeletal muscle disease. His mother had the same symptoms of chest tightness, dyspnoea, and abdominal distension and died of DCM at the age of 50 years. All available individuals with or without a positive history were evaluated via a full physical examination, chest radiography, echocardiography, and ECG. Medical records were reviewed in the case of deceased relatives. DCM was defined according to the criteria established by the World Health Organization/International Society and Federation of Cardiology Task Force on the Classification of Cardiomyopathy: a left ventricular end-diastolic diameter > 27 mm/m^2^ and an ejection fraction <40% or fractional shortening <25% in the absence of abnormal loading conditions, coronary artery disease, congenital heart lesions, and other systemic diseases.^[8]^

This work was approved by the ethics committee of the central hospital affiliated to Shandong First Medical University. Written informed consent was obtained from all participants before the study. The proband has provided informed consent for publication of the case.

### 2.2 Methods

#### 2.2.1 NGS

NGS was performed on the proband (II-1).

2 mL of peripheral blood was drawn into K_2_-EDTA tubes and stored at 4°C for a maximum of 24 hours. Genomic DNA was extracted from the blood using the TIANamp Blood DNA Kit (Tiangen Biotech Beijing Co. LTD., China). After DNA isolation, 1 μg genomic DNA was fragmented into about 200 bp lengths using the Covaris Acoustic System. The DNA fragments were then processed by end-repairing, A-tailing, adapter ligation, and library amplification. After the array capture, DNA libraries were sequenced on the Illumina Novaseq platform as paired-end 200 bp reads.

#### 2.2.2 Sanger sequencing

To validate the positive novel mutations identified via exome sequencing, Sanger sequencing was performed to confirm the presence or absence of these mutations in the proband, unaffected family members, and 50 unrelated healthy controls.

Specific PCR primers (forward primer 5’-TCTCCCCAACTCTGAAATGC-3’, reverse primer 5’- GGCTTAGGACAGTGCTTTGG-3’) were used for the amplification of exon 3 in the *TPM1* gene based on the reference sequences of the human genome from GenBank in NCBI (Gene ID: 7168). PCR cycling was performed on a DNA thermal cycler (Gene Amp 9700, Perkin-Elmer, USA) with the 2 × Hotstart Taq PCR Mastermix kit (Tiangen Biotech Beijing Co. LTD., China). In a 50 mL reaction mix, 300 ng of genomic DNA was used with 2.0 mL of each primer (10 mmol/L) and 25 mL of the 2 × PCR Mastermix. Genomic DNA was first denatured at 95°C for 5 minutes, followed by 25 cycles of 95°C for 30 seconds, 65°C (−0.6°C/cycle) for 30 seconds, 72°C for 40 seconds, and then 20 cycles of 95°C for 30 seconds, 50°C for 30 seconds, 72°C for 40 seconds. The PCR products were extended at 72°C for 10 minutes. The products were gel-purified with an agarose gel DNA purification kit (Tiangen Biotech Beijing Co. LTD., China), and the purified PCR products were sequenced using the forward and reverse primers. Automated sequencing was performed at both ends on an ABI 377 automatic sequencer. Mutations were interpreted according to the American College of Medical Genetics and Genomics (ACMG) recommended standard.^[9]^

#### 2.2.3 *In silico* analysis

After novel missense mutations were identified, *in silico* analyses were performed using 2 web-based tools, namely Polymorphism Phenotyping v2 (PolyPhen-2) and Sorting Intolerant from Tolerant (SIFT), to assess the deleterious effects of these newly detected mutations on the function of the TPM1. PolyPhen-2 (http://genetics.bwh.harvard.edu/pph2/) predicts the potential impact of an amino acid substitution on the structure and function of a human protein using straightforward comparative analyses of structural attributes of the mutated protein. PolyPhen-2 predicted3 possible outcomes of these mutations, based on scores ranging from 0 to 1, which were as follows: probably damaging, possibly damaging, or benign. SIFT (http://sift.bii.a-star.edu.sg/) is a sequences homology- based tool, which predicts the important amino acids that are conserved in a protein family. In this study, the results were expressed as SIFT scores, which were classified as damaging (0.00–0.05), potentially damaging (0.051–0.10), borderline (0.101–0.20), or tolerant (0.201–1.00).

## 3 Results

### 3.1 Clinical data

The proband's vital signs were stable on admission, and physical examination showed an enlarged left cardiac dullness border with a high heart rate (113 bpm). Laboratory tests were normal except for a slightly elevated neuron-specific enolase (24.11 ng/mL). The electrocardiogram showed ST-T wave changes and nodal tachycardia (Fig. 1B). Echocardiography revealed a left ventricular end-diastolic diameter of 33.6 mm/m^2^, an ejection fraction of 43.1%, and a fractional shortening of 21.7% (Fig. 1C).

### 3.2 Mutation detection

**Figure 2:**
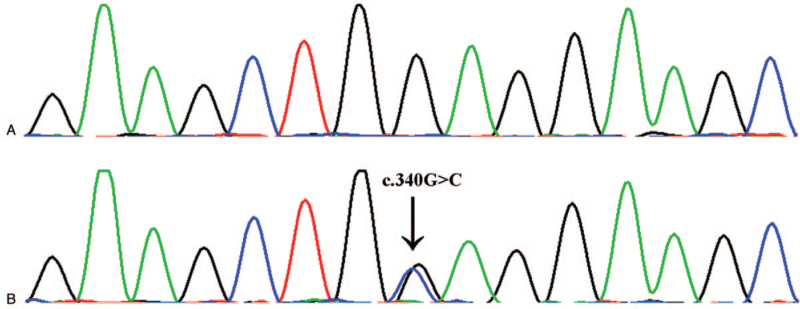
Sanger sequencing. (A) The healthy control. (B) The proband. Sanger sequencing shows a heterozygous missense mutation, c.340G > C in exon 3 of the *TPM1* gene of the proband. The arrow indicates the mutation site.

In this study, 19 variants in 18 genes were detected using NGS. We then excluded those variants with an allele frequency of more than 5% in the dbSNP database, 1000 human genome dataset, exome aggregation consortium (ExAC), and genome aggregation database (gnomAD). According to the detailed filtering criteria and analysis pipeline published before,^[10]^ a missense mutation, c.340G > C in exon 3 of the TPM1 gene, was revealed in the proband. This mutation was then confirmed by Sanger sequencing (Fig. 2). This heterozygous mutation results in the substitution of glutamic acid with glutamine (p.E114Q). No mutation at this site was found in available unaffected family members or 50 unaffected, unrelated healthy controls. According to the HGMD (http://www.hgmd.cf.ac.uk/docs/login.html), this heterozygous mutation is novel. Homology analysis of the p.E114 site in different animal species indicated that this amino acid is highly conserved (Table 1), which supports the possibility that this mutation is pathogenic.

**Table 1:** Comparison of sequences in the vicinity of TPM1 E114 from various species.

Species	Sequences in the vicinity of TPM1 E114
Homo sapiens	QERLATALQKLEEAEKAADESERGMKV
Mus musculus	QERLATALQKLEEAEKAADESERGMKV
Rattus norvegicus	QERLATALQKLEEAEKAADESERGMKV
Sus scrofa	QERLATALQKLEEAEKAADESERGMKV
Canis lupus familiaris	QERLATALQKLEEAEKAADESERGMKV
Cervus elaphus	QERLATALQKLEEAEKAADESERGMKV
Danio rerio	QERLATALQKLEEAEKAADESERGMKV
Canis lupus dingo	QERLATALQKLEEAEKAADESERGMKV

### 3.3 *In silico* analysis

The results of PolyPhen-2 and SIFT analyseis of c.340G > C in exon 3 of the TPM1 gene further provided conclusive evidence that this mutation is the cause of the clinical phenotype. This mutation was predicted to be probably damaging by Poly Phen-2 (score = 0.962) and deleterious by SIFT (score = 0.01).

Taken together, the diagnosis of DCM1Y was made by considering the clinical, echocardiographic, and ECG characteristics, as well as genetic testing. The patient was treated with metoprolol (23.75 mg, qd, po). He reported alleviation of symptoms at 1-year follow-up and is still on our follow-up list.

## 4 Discussion

DCM is the third most common cause of congestive heart failure and a major indication for heart transplantation.^[11,12]^ Patients with DCM are often first sent to the hospital for intermittent chest tightness upon physical or emotional stress. Conventionally, the diagnosis of DCM is established by echocardiography. In the present study, a missense mutation in a Chinese Han family with DCM is reported. The patients had typical features of DCM. Echocardiography of the proband showed a left ventricular end-diastolic diameter of 33.6 mm/m^2^, an ejection fraction of 43.1%, and a fractional shortening of 21.7%, consistent with the criteria for diagnosing DCM proposed by the World Health Organization/International Society and Federation of Cardiology Task Force on the Classification of Cardiomyopathy. Furthermore, using NGS and Sanger sequencing, we identified a novel missense mutation, c.340G > C in exon 3 of the *TPM1* gene, in this Chinese family. Based on the above genetic findings and clinical manifestations, a final diagnosis of DCM1Y was made in this family.

DCM1Y is characterized by severe progressive cardiac failure, resulting in death within the third to sixth decades of life in some patients. It is inherited in an autosomal dominant manner and caused by mutations in the *TPM1* gene on chromosome 15q22. The human *TPM1* gene contains 15 exons, among which exons 3, 4, 5, 7, and 8 are found in all *TPM1* variants, and exons 1a, 1b, 2a, 2b, 6a, 6b, 9a, 9b, 9c, and 9d are alternatively spliced. This gene encodes TPM1, a member of a family of actin-binding proteins implicated in the formation, stabilization, and regulation of cytoskeletal actin filaments. TPM1 plays a crucial role in regulating contraction by mediating the troponin complex calcium response to actin filaments in skeletal and cardiac muscles.^[13]^ The missense mutation, c.340G > C, revealed in this study, results in the substitution of glutamic acid with glutamine (p.E114Q). This substitution is expected to create a local increase in positive charge in a relatively negatively charged and highly conserved region of the molecule.

To date, about thirty mutations, including missense mutation, frameshift mutation due to deletion, and nonsense mutation (Table 2), have been reported in the *TPM1* gene for DCM in Pubmed, Embase, Web of Science, and the Human Gene Mutation Database (HGMD, http://www.hgmd.org/). The mutational screening of the *TPM1* gene is still far from complete. Identifying more novel mutations will provide more insights into the molecular pathogenesis of DCM. The mutation c.340G > C found in this research is a missense mutation. After searching the SNP database and the human gene mutation database, we found that this mutation was absent from these databases. This suggests that this Chinese Han family carries a novel heterozygous mutation.

**Table 2:** The summary of previously reported *TPM1* gene mutations.

Author, year	Neucleotide change	Coding effect	Position	Mutation type	References
Walsh, et al, 2017	c.2delT	M1RfsX11	Exon 1	Frameshift	^[14]^
Lakdawala, et al, 2012	c.23 T > G	M8R	Exon 1	Missense	^[15]^
Hershberger, et al, 2010	c.45 G > T	K15N	Exon 1	Missense	^[16]^
Hershberger, et al, 2010	c.67 G > C	E23Q	Exon 1	Missense	^[16]^
Walsh, et al, 2017	c.91G > A	A31T	Exon 1	Missense	^[14]^
Walsh, et al, 2017	c.97G > A	E33K	Exon 1	Missense	^[14]^
Hershberger, et al, 2010	c.107G > T	S36I	Exon 1	Missense	^[16]^
Walsh, et al, 2017	c.118G > T	Glu40X	Exon 2	Nonsense	^[14]^
Olson, et al, 2001	c.119G > A	E40K	exon 2	Missense	^[17]^
Olson, et al, 2001	c.161G > A	E54K	Exon 2	Missense	^[17]^
Pugh, et al, 2014	c.163G > A	D55N	Exon 2	Missense	^[18]^
van de Meerakker, et al, 2013	c.250G > A	D84N	Exon 3	Missense	^[19]^
Hershberger, et al, 2010	c.275T > C	I92T	Exon 3	Missense	^[16]^
Colpan, et al, 2017	c.333 G > T	Q111H	Exon 3	Missense	^[20]^
Pugh, et al, 2014	c.337C > G	L113V	Exon 3	Missense	^[18]^
Pugh, et al, 2014	c.341A > G	E114G	Exon 3	Missense	^[18]^
Walsh, et al, 2017	c.359C > T	A120V	Exon 3	Missense	^[14]^
Walsh, et al, 2017	c.368G > C	S123T	Exon 3	Missense	^[14]^
Pugh, et al, 2014	c.416A > T	E139V	Exon 4	Missense	^[18]^
Pugh, et al, 2014	c.423G > C	M141I	Exon 4	Missense	^[18]^
Van Spaendonck-Zwarts, et al, 2013	c.602C > T	T201M	Exon 6	Missense	^[21]^
Franaszczyk, et al, 2020	c.614A > G	K205R	Exon 6	Missense	^[22]^
Lakdawala, et al, 2012	c.632C > G	A211G	Exon 6	Missense	^[15]^
Lakdawala, et al, 2010	c.688G > A	D230N	Exon 7	Missense	^[23]^
Walsh, et al, 2017	c.695T > G	L232R	Exon 7	Missense	^[14]^
Hershberger, et al, 2010	c.715G > A	A239T	Exon 8	Missense	^[16]^
Pugh, et al, 2014	c.725C > T	A242V	Exon 8	Missense	^[18]^
Walsh, et al, 2017	c.734C > T	S245L	Exon 8	Missense	^[14]^
Hershberger, et al, 2010	c.830C > T	A277V	Exon 9	Missense	^[16]^
Wilson, et al, 2015	845C > G	T282S	Exon 9	Missense	^[24]^

## 5 Conclusion

Based on the results, we present evidence that p.E114Q mutation represents a novel *TPM1* mutation in a Chinese Han family with DCM1Y. Our data extend the mutation spectrum of the *TPM1* gene, provide new insights into the molecular basis for the pathogenesis of DCM and may aid early diagnosis.

## Acknowledgments

We thank the patients and their family members for their cooperation in this study. This work was supported by science and technology project of Jinan health commission (2020-3-02), and Shandong provincial natural science foundation (No. ZR2019MH043).

## Author contributions

**Data curation:** Yilong Man, Guangxin Wang.

**Formal analysis:** Meili Fan, Changying Yi, Guangxin Wang.

**Funding acquisition:** Changying Yi, Guangxin Wang.

**Investigation:** Yilong Man, Tianyu Yang, Peng Liu, Shiguang Liu.

**Project administration:** Changying Yi, Guangxin Wang.

**Resources:** Tianyu Yang, Peng Liu, Guangxin Wang.

**Software:** Meili Fan, Shiguang Liu.

**Supervision:** Changying Yi, Guangxin Wang.

**Writing – original draft:** Yilong Man, Meili Fan.

**Writing – review & editing:** Changying Yi, Guangxin Wang.
